# PPAR Research: Successful Launching and Promising Future

**DOI:** 10.1155/2009/543584

**Published:** 2009-07-30

**Authors:** Mostafa Z. Badr

**Affiliations:** Division of Pharmacology, School of Pharmacy, University of Missouri-Kansas City, 2411 Holmes, M3-115, Kansas City, MO 64108, USA

Since its inception a little over three years ago, PPAR Research has become a vibrant forum showcasing global effort in this ever-expanding field of research. Over this short period, more than 200 articles have appeared in the journal, both in regular and special issues. It is hoped that these articles have served as catalysts for new initiatives within the field. The following is a brief overview of the 12 special issues published, extracted from their respective editorials.

## 1. PPAR and Bone Metabolism

In this inaugural special issue of PPAR Research, a comprehensive overview of the role of PPAR-*γ* in the control of bone maintenance is presented. Articles published cover (1) PPAR-*γ*'s role in mesenchymal stem cell lineage allocation, (2) possible cross-talk with relevant nuclear receptors, (3) examination of PPAR-*γ* gene polymorphisms and bone mineral density in humans, (4) a role of PPAR-*γ* in bone loss due to skeletal disuse, (5) evidence that human bone is vulnerable to antidiabetic therapies using PPAR-*γ* agonists, the thiazolidinediones, and (6) evidence that the antiosteoblastic activity of PPAR-*γ* can be separated from its proadipocytic and antidiabetic activities. Also presented in this special issue is a novel hypothesis stipulating that PPAR-*γ* acts as a regulator of chondrocyte development and cartilage homeostasis.

## 2. PPARs and Obesity

This special issue begins with a review of key observations in human subjects harboring genetic variations in PPAR*γ* and a thorough overview of the metabolic effects of PPARs in genetically modified animal models. The interaction of PPARs with uncoupling proteins regulating energy expenditure is reviewed as are recent developments with RXR agonists. A closely related topic addressed is the molecular and physiological functions of PPAR coactivators and corepressors in relationship to adipocyte energy metabolism. In addition, the potential advantages of selective PPAR agonists are discussed. The intriguing possibility that PPARs may mediate effects of caloric restriction on longevity is also considered. Finally, evidence that PPARs may be interesting therapeutic targets to modulate obesity-induced inflammation is reviewed.

## 3. PPARs in Lung Pathophysiology and Disease

This special issue contains a comprehensive group of reviews and original investigations illustrating the pivotal role of PPARs in the regulation of multiple cellular events in lung pathogenesis. Such events include lung morphogenesis, the inhibition of the release of inflammatory mediators from lung immune and stromal/parenchymal cells in vitro, and dampening both inflammation and damage in animal models of acute lung injury (ALI), ischemia-reperfusion injury, and allergic airways inflammation. Also covered are lung tissue remodeling and the fibroproliferation that occur in chronic airways disease, ALI, pulmonary vascular disease, and pulmonary fibrosis. In addition, activation of key macrophage antimicrobial and reparative responses within the airspace is addressed, also data illuminating the central role of PPAR-*γ* in the regulation of critical aspects of lung tumor initiation, progression, and metastasis are summarized.

## 4. PPARs, RXRs and Stem Cells

This special issue compiles a mixture of excellent primary research manuscripts and comprehensive review articles from experts in the field covering the role of PPARs and RXRs in (1) murine embryonic stem cell differentiation and function, (2) hematopoietic stem cells, and (3) the classical role of PPAR*γ* as an adipogenic regulator in adipose tissue-derived stromal/stem cells. In addition, investigations examining bone marrow-derived mesenchymal stem cell (MSC) models provided novel findings relating to (1) the role of mystatin and GILZ on adipogenesis in response to PPAR ligands, (2) the transcriptomic response of MSCs to PPAR*γ* agonists, and (3) the role for farnesylation in modulating MSC adipogenesis are detailed. Finally, novel insights into the effect of PPAR*γ* during neural stem cell astroglial differentiation are presented.

## 5. PPARs/RXRs in Cardiovascular Physiology and Disease

This special issue contains a series of high-quality reviews starting with an examination of the effects of PPARs on lipoprotein metabolism, followed by a review evaluating the anti-inflammatory effects of PPARs in platelets, an emerging and important mechanism of the beneficial effects of these receptors. Next, an excellent discussion of the critical role that PPAR*α* and its transcriptional coactivator protein PGC-1*α* play in regulating energy metabolism and function of the myocardium is presented. Then there is a series of reviews focusing on the potentially beneficial effects of PPAR*γ* agonists on the cardiovascular system. Another review discusses the potential input of the hexarelin signaling pathway in regulating PPAR*γ* activity and its potential impact on cardio-metabolic disease. The pharmacogenomics of PPARs are also discussed in another review. Finally, the potential toxicity and adverse outcomes of PPAR agonism are summarized. In addition to these reviews, this special issue contains two original research reports. The first report discusses an association between PPAR*γ* gene polymorphisms and several cardio-metabolic indices, but found no link with cardiovascular morbidity or mortality. The second study details an important role for PGC-1*α* in post-natal metabolic maturation.

## 6. PPARs and RXRs in Male and Female Fertility and Reproduction

In this special issue, a broad picture of the potential role of the PPAR-RXR system in the reproductive axis is presented, raising new questions about the biological actions of this system in reproduction and its possible manipulation in treatments of fertility disorders. Presented reviews cover metabolic and immune contributions of PPARs to female fertility and reproduction to development of gametes and the preimplantation of embryos and placental development and function. Additional reviews discuss the roles of PPARs in the fetal origin of metabolic diseases and as mediators of phthalate-induced toxic effects in male and female reproductive tract. In conclusion, PPAR-*γ* as a potent target for prevention and treatment of human prostate and testicular cancer is reviewed.

## 7. PPARs in Eye Biology and Disease

This special issue contains a comprehensive group of reviews focused on the relationships of PPARs, choroidal neovascularization, inflammation, redox balance, and the perspective therapeutic potentials of PPAR modulators in eye diseases. Also, in this special issue, 3 papers are presented reviewing the current knowledge of the relationship between the PPAR isoforms, *α*, *β* and *δ*, and ocular angiogenesis with emphasis on age-related macular degeneration (AMD). An authoritative fourth paper addresses PPAR*γ* agonists as potential therapies for non-AMD proliferative retinopathies. Furthermore, presented are explorations of the roles of PPAR*γ* in microglial cell functions, therapeutic potentials of PPAR*γ* ligands on ocular diseases such as AMD, diabetic retinopathy, autoimmune uveitis and optic neuritis, and the role of PPAR*γ* in the breakdown of blood-retinal barrier. Also included is an extensive review of the role of lymphocytes in thyroid eye disease-related inflammation, which offered PPAR*γ* ligands as a potential therapeutic approach to this disorder. This special issue closes with a brief review of the cytoprotective effects of an endogenous PPAR*γ* agonist, 15d-PGJ_2_, on oxidative stress-induced retinal pigment epithelium cell death.

## 8. PPARs in Neuroinflammation

This special issue contains a series of reviews concerning the role of PPARs in neuroinflammatory diseases, as well as the potential role of PPARs in modulating CNS disorders including multiple sclerosis, Alzheimer's disease, spinal cord injury, stroke, traumatic brain injury, amyotrophic lateral sclerosis, and Huntington's disease. Also included in this special issue are reviews concerning the role of PPAR agonists in modulating the function of resident CNS microglia, and the molecular mechanisms by which PPARs regulate inflammatory signaling as related to CNS disease. This special issue also contains two original research reports. The first report provides a thorough investigation of the effects of PPAR-*γ* agonists in modulating the production of proinflammatory molecules by CNS microglia and astrocytes in response to distinct toll-like receptor ligands relevant to infections of the CNS. The second report investigates PPAR-*γ* agonist effects on amyloid beta-mediated microglial production of cytokines known to alter T-cell differentiation.

## 9. PPARs: A Double-Edged Sword in Cancer Therapy?

The title of this special issue reflects the unsolved paradoxical and mechanistically unclear effects of PPARs in cancer which can work both for and against this disease. This special issue contains 56 contributions which illuminate these disparate aspects of PPARs' role in cancer. They are organized in six different sections. Section 1 is a valuable collection of comprehensive overviews on PPARs in cancer and on the more established role of PPAR*γ* in cancer therapy. This is followed by Sections 2, 3, 4 with articles that discuss the following three key questions, respectively: (a) are PPARs friends or foes of tumors?, this basic dichotomy of PPARs' role in cancer has been fostered by numerous conflicting but unambiguous observations, (b) do PPARs modulate tumor cell-autonomous or non-cell autonomous (stromal) processes?, a question mirroring the emerging picture of neoplasia as a tissue disorder in which stromal cells play a critical role, and (c) are the cancer-related effects of PPAR ligands mediated by PPAR-dependent or independent “off-target” mechanisms? This special issue is closed with Section 5 which focuses on PPAR ligand-based cancer therapies and Section 6 which covers the potential molecular mechanisms of these ligands.

## 10. Development of Synthetic Modulators of PPARs: Current Challenges and Future Opportunities

Reports highlighting both challenges and opportunities in PPAR drug development are contained in this special issue. Clinical studies describing the effects of thiazolidinediones (TZD) on lipids, lipoproteins, and apolipoproteins are presented. Mechanism of TZDs in fluid retention and clinical data describing their effects on bone are also reviewed. In addition, the roles of PPARs in atherosclerosis and metabolic disorders as well as various strategies and technologies used in the identification and assessment of PPAR agonists are presented. Furthermore, effects of PPAR-*α* and PPAR-*γ* ligands on cysteinyl leukotriene production in mast cells as it relates to the development of potential anti-asthma medications are covered in this special issue. Species differences in plasma protein binding and in PPAR*γ* activation by a novel agonist, MBX-102, are described. Finally, the synthesis and SAR of subtype-specific PPAR agonists derived from a single 3,4-disubstituted phenylpropanoic acid “versatile template” scaffold is reported, along with a description of the development and safety profile of INT-131, a potent non-TZD selective PPAR modulator (SPPARM), a compound currently in Phase 2 clinical development.

## 11. Peroxisome Proliferator-Activated Receptor *δ*: A Target with a Broad Therapeutic Potential for Drug Discovery

The broad potential of PPAR-*δ* agonists for the treatment of metabolic disease is brought to light in this special issue. This includes a review on the regulation of the oxidative capacity of muscle by PPAR-*δ*, opportunities for the development of PPAR-*δ* agonists for the treatment of obesity, and finally the effect of PPAR-*δ* activation on vascular pathophysiological processes. The effects of PPAR-*δ* activation on cell proliferation and the risk of inducing cancer, an area of conflicting results and intense debate, is concisely reviewed in this special issue. Last but certainly not least, two additional interesting facets of PPAR-*δ* biology are presented. First, as PPAR-*δ* is expressed at high level in the brain, the potential neuroprotective role of PPAR-*δ* activation was detailed. Second, although the role of PPAR-*δ* in embryo implantation was recognized early on, the reproductive functions of PPAR-*δ* are still unclear. The latter issue is addressed in a review addressing potential applications of PPAR-*δ* ligands in assisted reproductive technology.

## 12. PPARs in Viral Disease

 This special issue contains an exciting array of valuable reviews examining the relationship between PPARs and various viral infections. First, the cross-talk between HIV infection and PPAR*γ* leading to the reported negative impact of this interplay on adipose tissue physiology is presented. Second, the effect of ART on adipocyte PPAR*γ* expression in vitro, in animal models, and in HIV-infected patients is summarized. In the third review, the results of 14 different clinical trials which evaluated if the PPAR*γ* thiazolidinedione agonists could be useful in the treatment of HAART-associated metabolic complications in HIV-infected patients are closely examined. In addition, there is a review which focuses on the potential role of PPAR*γ* in HIV-1-associated bone disease culminating with a provocative hypothesis stipulating a potential role for PPAR*γ* in the reduced bone mass associated with HIV-1 infection and treatment. As the liver is not only impacted by HIV but also by HBV and HCV, this organ is the focus of several excellent reviews in this special issue. First, the role that PPARs play in HIV infection, in terms of associated metabolic disorders, disease progression, coinfections with HBV or HCV and response to antiviral treatment is featured in a review. Further, experimental and clinical data suggesting that HCV may interfere with hepatic insulin signaling, possibly involving the downregulation of the PPAR*γ* are presented. Another review has summarized experimental and human studies showing a diminished expression and function of PPAR*α* and PPAR*γ* during HCV infection. An additional review discusses the potential of PPARs to modulate HBV transcription and replication, concluding with the proposal that modulating the PPAR*α*/RXR heterodimer may be an interesting therapeutic option to control HBV infection.

Examining the contents of these special issues reveals an intense interest in exploring new physiological roles of the PPARs and in the identification of new and improved PPAR agonist drugs. Nonetheless, safety issues raised for the currently marketed PPAR drugs should not be overlooked, and must be addressed. The Editorial Team of PPAR Research is committed to making the journal a premiere vehicle for the publication of the latest discoveries in these and other aspects of the field.

Mostafa Z. Badr

